# Demystifying Genetic Workup of Inborn Errors of Immunity

**DOI:** 10.1007/s11882-026-01303-3

**Published:** 2026-10-31

**Authors:** Batlle-Masó L., Gliagias V., Peng X.P.

**Affiliations:** 1 Translational Immunology Research Group, Immunology Division, Vall d’Hebron Research Institute (VHIR), Hospital Universitari Vall d’Hebron (HUVH), Barcelona, Catalonia Spain; 2 Jeffrey Modell Diagnostic and Research Center for Primary Immunodeficiencies, Barcelona, Catalonia Spain; 3 Department of Medicine, Division of Allergy and Immunology, Montefiore Medical Center, Bronx, NY USA; 4 Division of Pediatric Genetic Medicine, Department of Pediatrics, Children’s Hospital at Montefiore, Bronx, NY USA; 5 Division of Genomics, Department of Genetics, Albert Einstein College of Medicine, Bronx, NY USA; 6 New York Center for Rare Diseases, Bronx, NY USA

**Keywords:** Genetic testing, Inborn errors of immunity, Immune dysregulation, Genetically driven immune diseases, Molecular diagnosis

## Abstract

**Purpose of the review:**

Genetically driven immune diseases (GIDs) are germline or somatic errors of immunity, representing an expanding and diagnostically complex group of genetic disorders. While genomic advances have enhanced their detection, interpretation of genetic data remains a major challenge due to significant genotypic and phenotypic heterogeneity, incomplete knowledge about genotype–phenotype connections, and the diverse layers of mechanisms contributing to each patient’s specific expression of disease. In this review, we propose a clinically oriented framework for the genetic evaluation of patients with suspected GIDs.

**Recent findings:**

Using disorders featuring predominantly adaptive immune dysregulation as representative examples – hypomorphic defects in lymphocyte development (*RAG1*, *RAG2*), defects of central tolerance (*AIRE*), defects of peripheral tolerance (*FOXP3*), cytokine-mediated immune regulation (*IL2RA*, *IL2RB*), activation-induced cell death (*FAS*), and canonical NF-κB signaling (*CARD11* gain-of-function) – we provide a practical approach to the selection and use of clinically available genetic tests, emphasizing context-dependent strategies based on each patient’s clinical and immune findings. We also highlight key limitations of current genetic testing and emerging approaches aimed at improving diagnostic yield.

**Summary:**

This review provides a practical, stepwise, mechanism-informed framework for the genetic evaluation of patients with suspected GIDs that integrates clinical phenotyping with immune and genetic data. By emphasizing appropriate test selection, recognition of diagnostic limitations, and the use of complementary approaches in unresolved cases, this framework aims to support accurate molecular diagnosis and personalized management of patients with immune dysregulation.

## Introduction

Genetic defects affecting human immune development and regulation cause a diverse group of disorders historically known as primary immunodeficiencies (PID) and more recently as inborn errors of immunity (IEI) to encompass features beyond immunodeficiency such as adaptive and innate immune dysregulation. The term genetically driven immune disease (GID) has been proposed to further broaden the understanding of immune disorders as being caused by both germline and somatic genetic variants. Primary immune regulatory disorders (PIRDs), defined by the loss of immune homeostasis [1], represent one important subgroup within this broader spectrum. Although the principles discussed in this review apply broadly to all GIDs, we focus on PIRDs as representative examples, while also including selected GIDs where immune dysregulation is a prominent, though non-defining, clinical manifestation [2].

Major advances in genomics have transformed the diagnosis of GIDs [3], as declining sequencing costs and technological improvements have made genetic testing increasingly accessible. However, interpreting genomic data remains a major challenge. The number of genes associated with GIDs has increased dramatically, yet many patients remain undiagnosed [4, 5]. The reasons are multifactorial, including the challenges of GID biology itself, as well as limitations of technology, knowledge and access. In particular, we will highlight challenges stemming from our incomplete understanding of genotype–phenotype relationships, limiting variant analysis, triage and interpretation [4, 6]. Overcoming these challenges is crucial for achieving molecular diagnosis to inform clinical management, including the selection of targeted therapies or curative interventions.

**Fig. 1:**
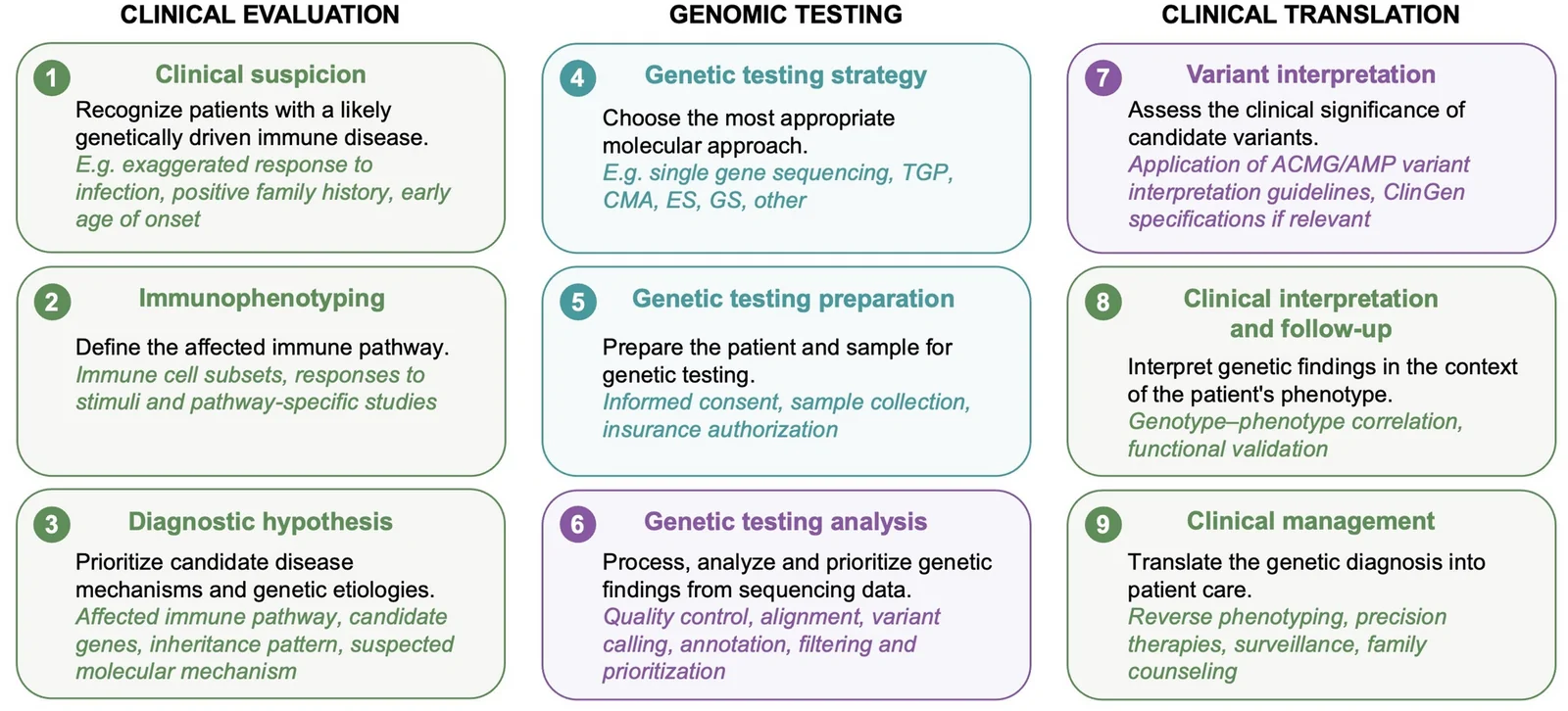
Framework for the genetic evaluation of patients with suspected genetically driven immune diseases (GIDs). The diagnostic evaluation of suspected GIDs is a multidisciplinary process integrating clinical assessment, immune phenotyping, genetic testing, variant interpretation, and clinical management. Colors indicate the primary responsibilities of each discipline. Green: clinical immunologists and treating physicians, who define the clinical question through detailed phenotypic and immunologic assessment and contextualize molecular findings within the clinical picture. Blue: clinical geneticists and genetic counselors, who select the appropriate testing strategy and coordinate test implementation. Purple: molecular laboratories, which perform genetic testing and interpret the variants. ACMG/AMP: American College of Medical Genetics and Genomics/Association for Molecular Pathology; CMA: chromosomal microarray, ES: exome sequencing, GS: genome sequencing; TGP: targeted gene panels

The genetic evaluation of suspected GIDs, including PIRDs, extends beyond sequencing alone and is best viewed as an iterative process integrating clinical assessment, immune phenotyping, and genomic analysis (Fig. 1). Suspicion of a GID prompts detailed immune characterization to identify the affected pathways and biological processes, helping to formulate a mechanism-based diagnostic hypothesis that guides genetic test selection and interpretation. Importantly, identifying a potentially disease-associated variant is not sufficient to establish its contribution to an individual’s phenotype; this must be assessed in the context of the clinical presentation, immunological findings, and other relevant genetic and environmental factors. Successful evaluation therefore requires close collaboration between clinical and diagnostic laboratory teams [7].

In this review, we propose a clinically oriented framework for the genetic evaluation of patients with suspected GIDs, using PIRDs and other GIDs where immune dysregulation is prominent, as representative examples. Rather than reviewing individual diseases in isolation, we use representative disorders to highlight major contributors to immune homeostasis and show how clinical, immunologic, and laboratory findings can be integrated to infer pathobiology and guide genetic testing.

## When to Consider Genetic Testing

An underlying PIRD often presents without significant infectious history, challenging recognition of these patients. Historically, the Jeffrey Modell Foundation (JMF) “10 warning signs” had been widely used as a clinical screening tool for GIDs [8]. Although these criteria remain useful in patients with recurrent, severe, unusual, or treatment-refractory infections [9], their sensitivity for GIDs is limited (approximately 45% in adults and 64% in children) [10] because of the focus on infection-based warning signs.

Indeed, up to 25% of patients with GIDs initially present with predominantly non-infectious manifestations [11, 12], including allergic, autoimmune, autoinflammatory, and lymphoproliferative manifestations [9]. The coexistence of these immune dysregulation features with recurrent or severe infections, malignancy, or syndromic features is a particularly important diagnostic red flag for an underlying GID [13]. Importantly, reduced penetrance and variable expressivity are often seen in GIDs [14], leading to under-recognition or missed diagnoses within affected families. For example, the same GID may have diverse ages of onset and clinical severities even within one family, so family members presenting later in life or with nonspecific or subtle manifestations may not be recognized until another family member with more obvious disease is diagnosed [15]. So even though a family history of recurrent infections, immune dysregulation, unexplained early deaths, consanguinity, or similarly affected relatives supports suspicion for an inherited disorder and may suggest potential inheritance patterns, the absence of a positive family history should not discourage genetic investigation, as many GIDs may arise *de novo*. Fundamentally, detailed longitudinal history-taking is essential before ordering genetic testing. The patient’s history from childhood and onward may contribute significantly to the differential, even if past presentations appear unrelated to the patient’s current clinical concerns (e.g. history of childhood infections and hospitalizations in an adult patient presenting with autoimmune cytopenias).

Characterization of the patient’s immune status sheds light on potential pathobiology and greatly aids the formulation of diagnostic hypotheses. Initial investigations may include complete blood counts, immunoglobulin levels, flow-based evaluation of different immune cell subsets, and assessment of vaccine responses [16]. Inflammatory marker trends such as CRP, ESR and serum amyloid A (SAA) can also be useful, as well as evidence of autoantibody production. In addition, more detailed innate and adaptive immune studies – via cytokine profiling, flow cytometry-based studies or RNA-based gene signatures – may help identify abnormalities in specific immune pathways, processes, or cell types. When interpreted together with other clinical and objective data, these studies may help one understand whether host-intrinsic immune dysregulation is driving the clinical concerns or arising as a consequence of other problems [17]. This distinction helps prioritize candidate genes for subsequent genetic testing while also providing critical biological context for the interpretation of genetic variants and strengthening of genotype–phenotype correlations.

**Fig. 2:**
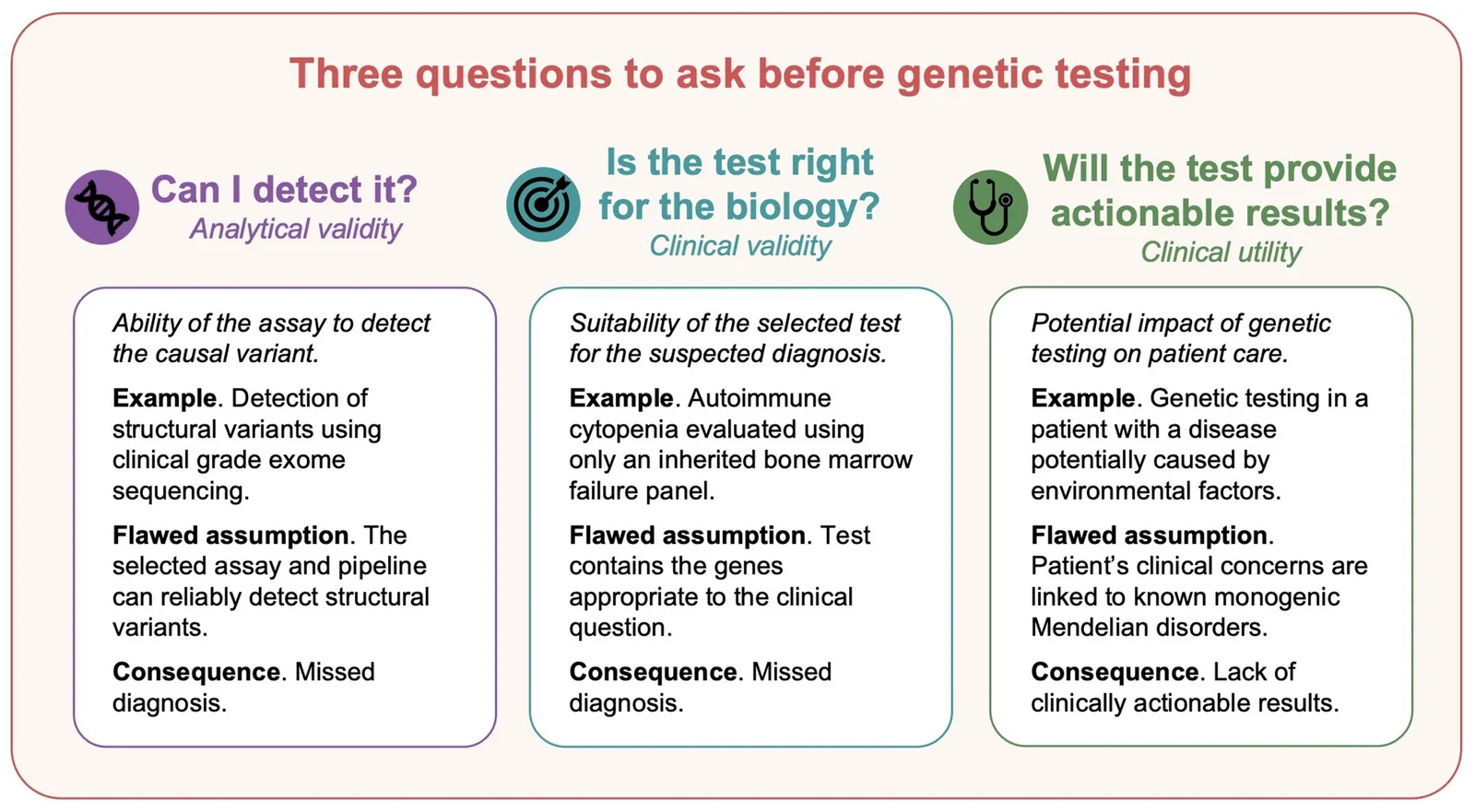
Three complementary principles guiding the selection of genetic testing. Genetic testing should be selected only when the selected platform is capable of detecting the expected class of pathogenic variants (analytical validity), the assay is appropriate for the suspected disease mechanism (clinical validity), and the result is expected to influence clinical management (clinical utility)

Once a PIRD is clinically suspected, the decision to pursue genetic testing should be guided by its analytical validity, clinical validity, and clinical utility (Fig. 2). Considering these principles separately can help avoid common pitfalls: using a platform unable to detect the variant type of interest (limited analytical validity); selecting an assay that does not interrogate the relevant genes or disease mechanisms (limited clinical validity); or requesting testing for conditions unlikely to have a genetic architecture amenable to dissection using current tools (limited clinical utility). Genetic testing should also be accompanied by basic pre-test counseling regarding the possible outcomes and their implications for the patient and their family.

Together, these clinical, familial, and immune findings not only support the suspicion of a PIRD but also help identify the source of inappropriate immune behaviors. The following representative clinical scenarios illustrate how characteristic combinations of clinical presentation and immune findings generate mechanism-based diagnostic hypotheses that guide molecular diagnostic testing strategy and interpretation.

## Recognizing Immunologic Patterns That Guide Genetic Testing

Once clinical suspicion for a PIRD has been established, the next challenge is recognizing patterns of immune dysregulation that point toward disruption of specific immune pathways. Although the phenotypic spectrum of PIRDs is broad and genotype–phenotype relationships are often complex, certain combinations of clinical and immune findings remain highly informative.

**Fig. 3:**
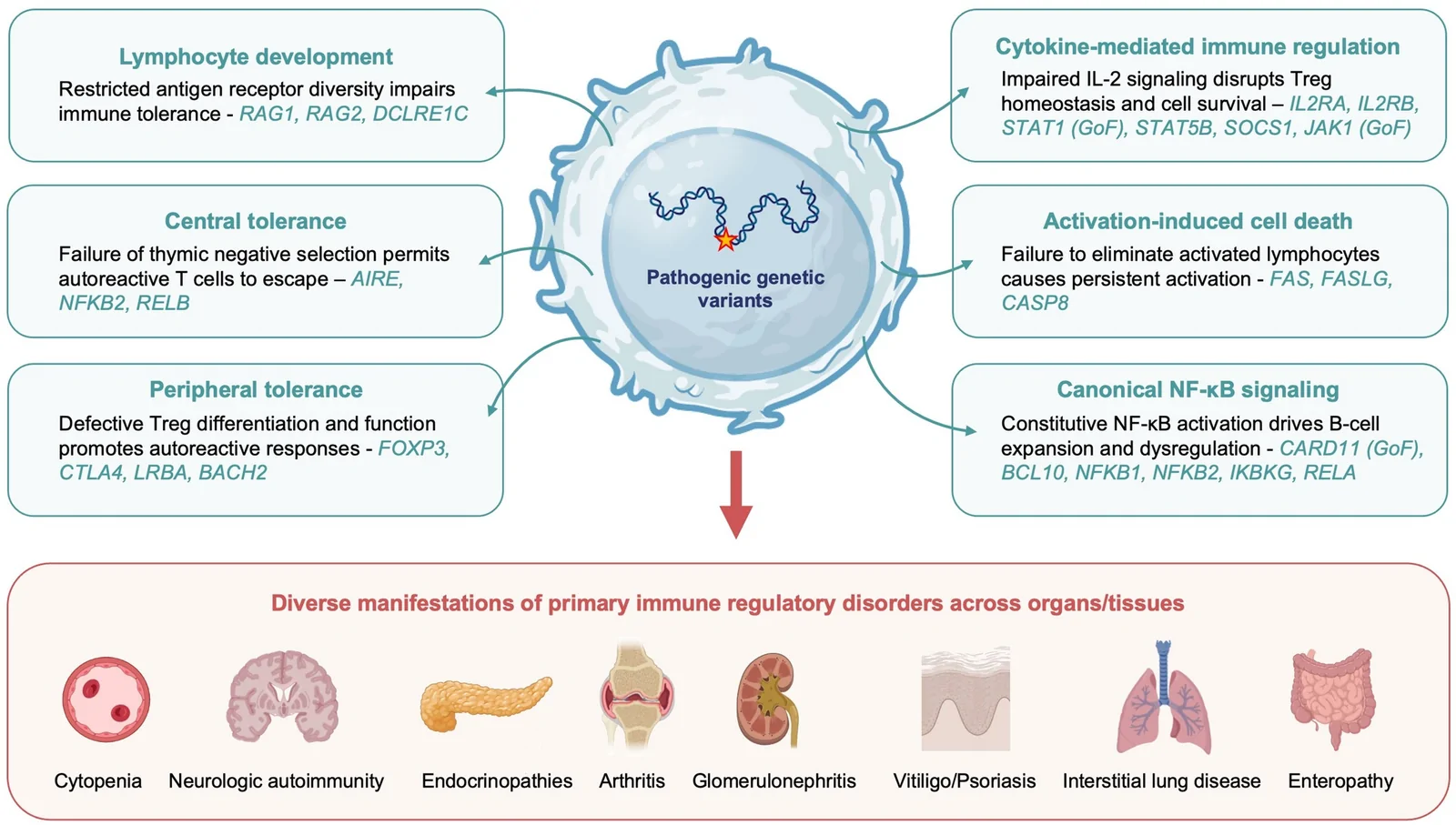
Representative pathogenic mechanisms underlying primary immune regulatory disorders. Pathogenic variants affecting key immune pathways and processes such as lymphocyte development, central and peripheral tolerance, cytokine signaling, activation-induced cell death, and canonical NF-κB signaling may produce characteristic patterns of immune dysregulation that ultimately manifest as diverse autoimmune and inflammatory diseases across multiple organ systems. Representative genes are provided to illustrate each mechanistic group rather than an exhaustive list of associated disorders

The following clinical scenarios illustrate prototypical forms of immune dysregulation rather than individual gene defects. Although each case focuses on one well-characterized disorder, similar clinical and immunologic phenotypes may arise from pathogenic variants affecting other genes within the same pathway summarized in Table 1; Fig. 3. It is important to note that these examples do not represent the full spectrum of PIRDs, and that additional clinically relevant mechanistic groups include activated PI3Kδ syndrome and related disorders of PI3K signaling (e.g., *PIK3CD*, *PIK3R1*, *PTEN*), primary/familial hemophagocytic lymphohistiocytosis (HLH) caused by defects in cytotoxic lymphocyte degranulation (e.g., *PRF1*, *UNC13D*, *STX11*, *STXBP2*, *RAB27A*), and other disorders of dysregulated cytokine signaling. Although not included in this review, these disorders are increasingly recognized because of the availability of mechanism-based therapies, highlighting the importance of pathway-oriented genetic diagnosis.

**Table 1:** Representative pathobiological groups of primary immune regulatory disorders (PIRDs), associated genes, characteristic immune phenotypes, and relevant diagnostic tips

Pathobiological group	Representative genes	Characteristic immune phenotype	Relevant diagnostic tips
Hypomorphic defects in lymphocyte development	*RAG1*,* RAG2*,* DCLRE1C*	Combined immunodeficiency with immune dysregulation (e.g. granulomatous disease, severe atopy, autoimmunity).	Hypomorphic variants may escape newborn screening because residual T-cell development can generate detectable TRECs; interpretation should consider residual recombinase activity.
Defects in central tolerance	*AIRE*,* NFKB2*,* RELB*	Organ-specific autoimmunity, chronic mucocutaneous candidiasis, autoimmune endocrinopathies.	*AIRE* variants may be biallelic (recessive) or, less commonly, monoallelic with dominant-negative effect, with clinical expression ranging from classic APECED triad to incomplete/atypical presentations.
Defects in peripheral tolerance (Tregopathies)	*FOXP3*,* CTLA4*,* LRBA*,* BACH2*	Early-onset multisystem autoimmunity (enteropathy, endocrinopathy, dermatitis, cytopenias).	Promoter or copy-number variants (e.g., *FOXP3*, *CTLA4*, *LRBA*) may not be detected by routine variant-calling pipelines; careful assessment of non-coding and structural variants may be required.
Defects in cytokine signaling (JAK–STAT pathway)	*IL2RA*,* IL2RB*,* STAT1* (GoF), *STAT5B*,* SOCS1*,* JAK1* (GoF)	Autoimmunity with recurrent infections, lymphoproliferation, enteropathy, and impaired immune regulation.	Accurate interpretation requires distinguishing gain- from loss-of-function mechanisms of disease; somatic mosaicism has been reported for selected genes (e.g., *JAK1*).
Defects in activation-induced cell death	*FAS*,* FASLG*,* CASP8*	Chronic non-malignant lymphoproliferation with autoimmune cytopenias.	Somatic *FAS* variants may be missed by standard germline sequencing pipelines; analysis of sorted lymphocyte populations or high-depth sequencing may improve detection.
Defects in canonical NF-κB signaling	*CARD11*,* BCL10*,* NFKB1*,* NFKB2*,* IKBKG*,* RELA*	B-cell lymphocytosis, atopy, recurrent infections, humoral immunodeficiency, immune dysregulation.	C-terminal *NFKB1*/*NFKB2* variants are hard to interpret functionally, as they may differentially affect p105/p50 (NF-κB1) vs. p100/p52 function (NF-κB2); assessment should evaluate both processing and transcriptional activity.
PI3K signaling disorders	*PIK3CD* (GoF), *PIK3R1* (LoF), *PTEN*	Recurrent infections, lymphoproliferation, herpesvirus susceptibility, autoimmunity.	Interpretation requires consideration of both activating and inactivating mechanisms within the PI3K pathway.
CTL/NK-cell degranulation defects (familial HLH)	*PRF1*,* UNC13D*,* STX11*,* STXBP2*,* RAB27A*	Hyperinflammation, hemophagocytic lymphohistiocytosis, severe viral infections.	Structural and deep intronic variants (particularly in *UNC13D*) may not be detected by routine ES and may require complementary molecular approaches.

Prototypic disorders correspond to the representative clinical examples discussed in the text and illustrate broader pathobiological groups rather than individual gene–disease relationships. Representative genes are illustrative rather than exhaustive. Relevant diagnostic tips highlight molecular mechanisms that may complicate variant detection or interpretation

*APECED*, autoimmune polyendocrinopathy-candidiasis-ectodermal dystrophy, *GoF* gain-of-function, *HLH* hemophagocytic lymphohistiocytosis, *LoF*, loss of function, *TRECS* T-cell receptor excision circles

### Hypomorphic Defects in Lymphocyte Development Exemplified By RAG1 and RAG2 Deficiency

The recombination-activating genes *RAG1* and *RAG2* encode the endonuclease complex required for V(D)J recombination and the generation of diverse T- and B-cell antigen receptor repertoires. Nullomorphic *RAG1/2* variants abolish V(D)J recombination and cause T and B cell negative severe combined immunodeficiency. In contrast, hypomorphic variants that retain partial recombinase activity permit residual T- and B-cell development, resulting in a broad spectrum of disease in which immune dysregulation may predominate over severe immunodeficiency. Defective central and peripheral immune tolerance in these patients can manifest as autoimmunity, granulomatous inflammation, and severe atopy, often accompanied by recurrent infections [18].

Representative case: A 14-year-old girl, born to consanguineous parents, presents with granulomatous skin lesions, autoimmune hemolytic anemia, and recurrent sinopulmonary infections. She was previously diagnosed with “idiopathic CD4^+^ lymphopenia.” Evaluation reveals low but detectable T and B cells, a restricted T-cell receptor repertoire, elevated IgE, and multiple autoantibodies.

The combination of immune dysregulation with lymphopenia and recurrent infections should raise suspicion for hypomorphic defects affecting lymphocyte development. Unlike patients with null *RAG1/2* alleles, who are typically identified by newborn screening through absent T-cell receptor excision circles (TRECs) [19], individuals with hypomorphic variants may escape early detection and present later with organ- or tissue-specific autoimmunity, granulomatous inflammation, and/or severe atopy. Recognition of this phenotype should prompt genetic testing that includes *RAG1* and *RAG2*, among other genes affecting lymphocyte development and V(D)J recombination. Early molecular diagnosis facilitates timely hematopoietic stem cell transplantation (HSCT) before irreversible organ damage develops [20].

Hypomorphic *RAG1/2* deficiency represents one example of impaired lymphocyte development. Similar phenotypes may result from defects affecting V(D)J recombination and DNA repair, such as *DCLRE1C*, as well as other disorders impacting lymphocyte development.

### Defects in Central Tolerance Exemplified By APECED (AIRE Deficiency)

Central tolerance eliminates self-reactive T cells during thymic development. The autoimmune regulator (AIRE) promotes the expression of tissue-restricted antigens by medullary thymic epithelial cells, enabling negative selection of autoreactive T cells before they enter the peripheral immune repertoire. Pathogenic *AIRE* variants disrupt this process, allowing autoreactive T cells to enter the peripheral repertoire, resulting in a characteristic pattern of organ-specific autoimmunity [21].

Representative case: A 6-year-old girl presents with recurrent oral thrush refractory to topical antifungal therapy, followed by hypoparathyroidism and later adrenal insufficiency. Over subsequent years, she develops alopecia, enamel hypoplasia, and vitiligo.

The combination of chronic mucocutaneous candidiasis with autoimmune endocrinopathies should prompt evaluation for autoimmune polyendocrinopathy-candidiasis-ectodermal dystrophy (APECED) [22]. Autoantibodies against IL-17 A, IL-17 F, and IL-22 can support the APECED diagnosis, as they arise due to the underlying defect in central tolerance. However, the diagnosis confirmation relies on identification of biallelic *AIRE* loss-of-function variants, while dominant-negative variants in the first plant homeodomain (PHD1) zinc finger and SAND domain lead to later-onset, milder phenotypes with reduced penetrance [23]. Early molecular confirmation enables surveillance for additional autoimmune manifestations, guides appropriate endocrine management, and facilitates genetic counseling.

APECED is the prototypical disorder of defective central tolerance. Although AIRE deficiency is the most common cause, rare *NFKB2* and *RELB* defects can also cause thymic stromal abnormalities and reduced or absent AIRE-expressing medullary thymic epithelial cells [24]. Thus, similar phenotypes may arise from defects affecting other components of central tolerance. Of note, in rare thymoma patients, autoantibodies are themselves neutralizing and pathogenic, directly producing chronic mucocutaneous candidiasis and thereby phenocopying inherited defects of Th17 immunity in patients without any germline lesion [25].

### Defects in Peripheral Tolerance Exemplified By IPEX Syndrome (FOXP3 Deficiency)

Peripheral tolerance is maintained primarily by FOXP3⁺ regulatory T (Treg) cells, which suppress autoreactive T cells and maintain immune homeostasis. Loss of FOXP3 disrupts Treg development and function, permitting pathogenic effector T-cell activation and resulting in T-cell-mediated tissue injury and humoral autoimmunity.

Representative case: A 3-month-old boy presents with intractable diarrhea, failure to thrive, eczematous dermatitis, and neonatal-onset diabetes mellitus. Laboratory studies demonstrate elevated serum IgE, eosinophilia, and Coombs-positive hemolytic anemia. His maternal uncle reportedly had childhood-onset eczema and later developed autoimmune thyroiditis without enteropathy or diabetes.

Early-onset enteropathy, endocrinopathy, eczema, and autoimmune cytopenias are characteristic of immune dysregulation, polyendocrinopathy, enteropathy, X-linked (IPEX) syndrome. As for most GIDs, marked intrafamilial phenotypic variability has been reported [26]. Although flow cytometric assessment of FOXP3 expression may provide supportive information, normal or reduced expression neither confirms nor excludes the diagnosis and should be interpreted cautiously. Patients with an IPEX-like phenotype should undergo comprehensive genetic testing that includes *FOXP3*,* IL2RA*,* IL2RB*,* CTLA4*,* LRBA*, and other genes associated with Treg development and function. Establishing the diagnosis is essential because HSCT remains the only curative therapy, while immunomodulatory agents such as sirolimus may help control disease before transplantation [27].

IPEX syndrome represents prototypical Tregopathy. Similar defects in peripheral tolerance may result from pathogenic variants in *CTLA4*, *LRBA*, and *BACH2*.

### Defects in Cytokine-Mediated Immune Regulation Exemplified By IL2RA and IL2RB Deficiency

The interleukin-2 receptor (IL-2R) is a heterotrimeric complex composed of the α (CD25), β (CD122), and common γ (CD132) chains [28]. IL-2 signaling is essential for peripheral tolerance by promoting Treg cell development and homeostasis, while also supporting effector T-cell function [29].

Representative case: A 6-month-old infant presents with severe enteropathy, eczema, recurrent respiratory infections, and autoimmune diabetes. Flow cytometry demonstrates absent CD25 expression on activated CD4⁺ T cells. A second patient presents in infancy with severe CMV infection, diffuse lymphadenopathy, bowel inflammation, hypergammaglobulinemia, and dysregulated NK-cell development.

*IL2RA* (CD25) deficiency impairs formation of the high-affinity IL-2 receptor, resulting in defective Treg function and an IPEX-like phenotype characterized by enteropathy, dermatitis, immune cytopenias, and recurrent infections. Complete absence of CD25 expression is a useful screening clue but is not specific for *IL2RA* deficiency and may occur in other immune dysregulation disorders [30], infection, drug exposure, or technical artifacts. Abnormal CD25 expression should therefore be interpreted alongside the clinical phenotype, additional immunologic studies, and comprehensive genetic testing rather than directing single-gene sequencing alone.

By contrast, *IL2RB* (CD122) deficiency disrupts signaling through both IL-2 and IL-15 [31], leading not only to impaired peripheral tolerance but also to defective NK-cell development and CD8⁺ T-cell homeostasis [32]. Patients typically present with severe immune dysregulation, including autoimmunity, enteropathy, lymphadenopathy, and hypergammaglobulinemia, often accompanied by severe viral infections, particularly CMV. Recognition of these phenotypes should prompt genetic testing that includes *IL2RA*,* IL2RB*, and other genes involved in IL-2 signaling and Treg biology. Identification of the molecular defect is important because both disorders are potentially curable with HSCT.

*IL2RA* and *IL2RB* deficiencies exemplify disorders of dysregulated cytokine signaling. Similar immune dysregulatory phenotypes have been described in patients with variants affecting the JAK–STAT pathway, including *STAT1* (gain-of-function), *STAT5B*, *SOCS1*, and *JAK1* (gain-of-function).

### Defects in Activation-Induced Cell Death Exemplified By ALPS

Following immune activation, lymphocytes are eliminated through FAS-mediated apoptosis, thereby contributing to the resolution of immune responses and maintaining immune homeostasis. Defects in this pathway impair activation-induced cell death, resulting in chronic non-malignant lymphoproliferation, autoimmunity, and an increased risk of lymphoma [33].

Representative case: A 12-year-old boy presents with a history of resolved Coombs-positive autoimmune hemolytic anemia at age 4, followed by recurrent immune thrombocytopenia at age 9, and recently emerging cervical lymphadenopathy and splenomegaly despite the absence of active infectious symptoms. Laboratory evaluation demonstrates an expanded population of TCRαβ⁺CD4⁻CD8⁻ double-negative T (DNT) cells.

The combination of chronic non-malignant lymphadenopathy, splenomegaly, and autoimmune cytopenias should be suggestive of autoimmune lymphoproliferative syndrome (ALPS). Although expansion of DNT cells supports the diagnosis, it is not specific and may also occur in other immune dysregulation disorders, including disorders of Treg or CBM function (see below) and JAK-STAT gain of signaling [34]. Characterization of FAS-controlled DNT subsets identified by CD38 and CD45RA co-expression may improve diagnostic specificity and help prioritize sequencing of *FAS* pathway genes [35]. Molecular diagnosis guides treatment, including consideration of sirolimus as first-line corticosteroid-sparing therapy, avoidance of unnecessary splenectomy with its associated infection risks, and surveillance for lymphoma.

ALPS is the prototypical disorder of defective activation-induced cell death. Similar phenotypes may result from pathogenic variants affecting other components of the FAS apoptotic pathway, including *FASLG* and *CASP8*.

### Defects in Canonical NF-κB Signaling Exemplified By BENTA Disease (CARD11 Gain-of-Function)

CARD11 is a scaffold protein of the CARD11–BCL10–MALT1 (CBM) signalosome that links antigen receptor signaling to canonical NF-κB activation. Germline gain-of-function *CARD11* variants result in constitutive NF-κB signaling, driving expansion of immature and naïve B cells while inducing functional T-cell anergy. The resulting phenotype, known as B-cell Expansion with NF-κB and T-cell Anergy (BENTA) disease, is characterized by persistent polyclonal B-cell lymphocytosis, impaired humoral immunity, recurrent infections, and immune dysregulation [36]. BENTA is typically inherited in an autosomal dominant manner and *de novo* cases have been reported. Moreover, rare autosomal recessive forms caused by biallelic *CARD11* variants have been described and it is also important to note that CARD11-related disease spans a broader phenotypic spectrum, with heterozygous dominant negative variants causing CADINS (CARD11-associated atopy with dominant inheritance and an impaired NF-κB signaling phenotype, and autosomal recessive loss-of-function variants causing combined immunodeficiency [37].

Representative case: A 5-year-old boy presents with recurrent upper respiratory infections, progressive splenomegaly on exam, and persistent lymphocytosis on routine blood work. Laboratory evaluation demonstrates marked expansion of transitional and naïve B cells, reduced class-switched memory B cells, hypogammaglobulinemia with poor vaccine responses, and impaired T-cell proliferative responses.

Persistent polyclonal B-cell lymphocytosis with expansion of transitional and naïve B cells, together with hypogammaglobulinemia and impaired humoral responses should raise suspicion for BENTA disease and prompt genetic testing that includes *CARD11*. Molecular diagnosis is clinically important because affected individuals are at increased risk of B-cell lymphoma. Acute HLH-like diatheses have also been reported [38]. Recognition of the molecular diagnosis guides immunoglobulin replacement, management of immune dysregulatory complications, and appropriate long-term surveillance.

BENTA disease represents one manifestation of dysregulated canonical NF-κB signaling. Related phenotypes that can lead to NF-κB-related immune dysregulation include pathogenic variants in *BCL10*, *NFKB1*, *NFKB2*, *IKBKG*, and *RELA*.

## Selection of Genetic Testing Strategies

Although by no means exhaustive for all PIRD pathobiologies, our examples above illustrate how recognition of clinical and immunophenotypic patterns may help hone suspicion for a PIRD and even suggest specific pathways and processes to consider on the genetic differential. The challenge then shifts toward selecting an appropriate genetic testing strategy. High throughput short-read sequencing (SRS) now underpins most diagnostic workflows in this field, but in practice, the choice between locus-specific approaches, targeted gene panels (TGPs), exome sequencing (ES), and genome sequencing (GS) remains highly context-dependent. Clinical data, laboratory findings, urgency, family preferences, available resources, and logistical considerations all contribute to this decision.

In many cases, TGPs represent a logical first step. This is particularly true when the aggregate data strongly points toward a discrete and recognizable group of pathobiologies; for example, the combination of infection-induced high fevers, transaminitis, acute-onset neurological changes, disproportionately high CXCL9 level relative to IL-18, and abnormal CD107a mobilization by NK cells would strongly suggest the ordering of a dedicated primary HLH gene panel. TGPs offer practical advantages: they are relatively inexpensive, provide high and uniform sequencing depth and may facilitate timely diagnosis depending on the clinical setting and laboratory workflow. Some panels will also offer valuable orthogonal testing for variants that are difficult-to-detect on SRS, such as the recurrent inversion and non-coding variants associated with *UNC13D*-related HLH [39]. When appropriately selected and interpreted in the context of the clinical phenotype, they can also simplify variant interpretation by restricting analysis to genes with established disease associations [40]. For patients with a well-defined clinical phenotype, this remains an effective first-line strategy in many healthcare systems, particularly where access to ES or GS is limited [4].

Broader sequencing approaches become preferable when the phenotype is atypical, multisystem, or evolving—or when the genetic architecture of disease remains incompletely defined. Patients with strong clinical suspicion but negative panel testing should be considered for ES or GS. An increasingly adopted strategy is to obtain TGPs on an exome or genome backbone so that initial analysis can be restricted to a phenotype-driven gene list [41]. One can then reflex to broader analysis if no diagnosis is established. This strategy combines the flexibility of broad sequencing with the interpretive advantages of a targeted analysis.

By interrogating the coding regions of nearly all genes, ES enables comprehensive analysis of clinically established disease genes without being restricted to predefined panels. At the same time, it facilitates periodic reinterpretation as new disease genes become clinically validated [42]. GS provides an even more comprehensive assessment and may be considered when ES is nondiagnostic, particularly because it enables detection of structural, copy-number, repeat expansion, and non-coding variants that are not adequately captured by ES. Whenever feasible, trio-based sequencing including parental samples further improves diagnostic yield by facilitating detection of *de novo* variants and clarifying variant segregation (phasing).

Beyond their diagnostic utility, research-based ES and GS also support identification of novel candidate genes, although establishing new gene–disease associations requires dedicated research frameworks and appropriate ethical oversight. These research efforts have led to the discovery of previously unrecognized disease genes [43], and expanded the phenotypic spectrum of established disorders such as *JAK1*-associated disease [44], and revealing novel allelic mechanisms in genes already associated with GIDs, including *TRAF3* haploinsufficiency [45] and *PLCG1* gain-of-function variants [46].

On the horizon, long-read sequencing (LRS) platforms are beginning to address additional limitations of SRS platforms, particularly phasing, repeat expansions, complex structural variation, and regions of the genome with high sequence homology or repetitive sequence. This is especially relevant for immune genes with highly homologous pseudogenes such as *NCF1* and *IKBKG*, although clinical use of LRS for routine genetic diagnostic testing is only just emerging [47].

Taken together, these approaches should be viewed as complementary rather than hierarchical, and the optimal strategy depends on phenotype complexity, diagnostic urgency, expertise availability, and healthcare resources. They should be revisited as the clinical phenotype evolves and new gene-disease relationships become clinically validated.

## When Genetic Testing May Not Be Necessary

While genetic testing has become increasingly accessible, indiscriminate testing is unlikely to improve diagnostic yield and may instead complicate interpretation through the identification of incidental or uncertain findings. Careful assessment of pre-test probability therefore remains essential.

A central question in the assessment of any patient is whether the magnitude or nature of the immune response is appropriate to the inciting stimulus. For example, recurrent but self-limited upper respiratory tract infections in an otherwise healthy child attending daycare more likely represent expected physiological response to repeated pathogen exposure rather than evidence of an intrinsic immune defect [48]. Likewise, infections that are readily explained by local or environmental factors, such as cigarette smoke exposure or structural abnormalities (e.g., adenoidal hypertrophy), have a low pre-test probability for an underlying GID. In contrast, genetic investigation is most informative when the inflammatory or infectious phenotype is unexpectedly severe, persistent, multisystem, or otherwise disproportionate to the triggering stimulus, suggesting underlying dysregulated immunity rather than a normal physiological response [49].

Patients without clinically meaningful abnormalities on baseline immunologic assessment—including immunoglobulin levels, lymphocyte counts and differentiation, and vaccine responses interpreted in the appropriate clinical context—are unlikely to benefit from genetic testing for the indication of mild, self-limited recurrent sinusitis or otitis media alone. Similarly, isolated autoimmunity such as Hashimoto’s thyroiditis or type 1 diabetes responsive to standard therapy, in the absence of cytopenias, lymphoproliferation, recurrent infections or other immune-mediated clinical concerns, generally does not warrant broad genetic investigation. Conversely, treatment-refractory or multisystem autoimmunity, particularly when accompanied by recurrent infections or laboratory evidence of immune dysfunction, substantially increases the likelihood of underlying GID.

## Limitations of Genetic Testing

Even when genetic testing is appropriately indicated, the absence of a definitive molecular diagnosis does not exclude an underlying PIRD. Several clinical, biological, and technical factors may limit the sensitivity of standard sequencing approaches. Importantly, the diagnostic yield of genetic testing depends heavily on the availability of accurate and detailed clinical and immune data. Incomplete or inaccurate phenotypic characterization may lead to inappropriate test selection or suboptimal variant prioritization, particularly for ES or GS. Examples of flawed assumptions and diagnostic tips are detailed in Table 1; Fig. 2.

Structural variants, including deletions, duplications, inversions, and complex rearrangements, remain an important cause of false-negative genetic testing because their detection varies across sequencing platforms and bioinformatic pipelines. These variants are increasingly recognized in PIRDs, including disorders caused by pathogenic variants in *DOCK8* [50], *IKZF1* [51], *CTLA4* [52] and the aforementioned *UNC13D* [39]. Although dedicated calling algorithms can infer many structural variants from SRS data, sensitivity remains variable depending on sequencing coverage, genomic architecture, and analytical methods [53]. When structural variation is strongly suspected, complementary approaches such as chromosomal microarray (CMA), multiplex ligation-dependent probe amplification (MLPA), or optical genome mapping may improve diagnostic yield.

Pathogenic variants in non-coding regions may also be missed by strategies that focus sequencing or variant interpretation on coding regions [54]. These are increasingly recognized in GIDs and include variants affecting splicing, transcriptional regulation, and other non-coding regulatory elements [55]. In addition, certain genomic loci remain technically challenging for short-read mapping and alignment because of pseudogenes, segmental duplications, or repetitive sequence. In these settings, complementary targeted methods—including Sanger sequencing, PCR-based assays, MLPA, and CMA—may provide better analytical performance. However, bioinformatic advances alone can also enhance diagnostic yields. For example, nested PCR has traditionally been required for *NCF1* because of pseudogene interference [56], but improved computational algorithms can now reliably detect recurrent pathogenic variants from SRS data [56, 57].

Another important consideration is the possibility of somatic mosaicism, which has been associated with a number of immunoregulatory and inflammatory disorders. In conditions such as somatic ALPS, for example, pathogenic *FAS* variants are enriched in the DNT αβ cell population and may present at low variant allele fractions in peripheral blood DNA. Although ES and GS can detect somatic variants, their sensitivity is strongly dependent on sequencing depth, coverage uniformity, variant allele fraction, and the bioinformatic pipeline used. Dedicated somatic variant callers, combined with high-depth sequencing, can improve detection sensitivity [58]. In selected cases, ultra-deep sequencing, digital droplet PCR, molecular inversion probes, or sequencing of sorted cell populations can further increase diagnostic yield [59, 60]. Recognition of mosaicism has important implications for prognosis, recurrence risk assessment, and therapeutic decision-making.

Finally, acquired anti-cytokine autoantibodies represent a relevant non-genetic cause of immune dysfunction that can phenocopy GEI. If these autoantibodies have neutralizing activities, they may disrupt specific immune signaling pathways and produce secondary immunodeficiency. For example, anti-IFN-γ autoantibodies can cause an acquired defect of the IL-12/IFN-γ axis associated with disseminated nontuberculous mycobacterial infections [61], closely resembling genetic defects in this pathway. Therefore, when the clinical phenotype defects in a specific signaling pathway but genetic testing is unrevealing, evaluation for acquired anti-cytokine autoantibodies should be considered as a potential alternative explanation.

## Variants of Uncertain Significance

Even if variants are appropriately detectable, their interpretation remains a major challenge. The ACMG/AMP framework [62] classifies variants into five categories—pathogenic, likely pathogenic, uncertain significance (VUS), likely benign, and benign—based on different evidence streams, including phenotypic, population, computational, functional, and familial data. Moreover, for some diseases this framework has subsequently been refined by ClinGen Variant Curation Expert Panels (VCEPs) that develop gene- and condition-specific guidelines for applying individual criteria. However, use of these more disease paradigm-specific criteria may not always lead to fewer VUSes.

A VUS indicates that the available evidence is currently insufficient to determine whether a variant is disease-causing or benign. Importantly, it should not be used on its own to establish or exclude a molecular diagnosis, or to guide clinical management. Nevertheless, a VUS identified in a gene with known disease association strongly compatible with the patient’s phenotype encourages further investigation. Rather than relying on a single piece of evidence, variant interpretation should consider multiple complementary lines of evidence and be revisited over time as new information becomes available [63].

In silico prediction tools can provide an initial assessment of the potential effect of a variant. Tools such as CADD [64] and REVEL [65] can help assess the potential deleteriousness of genetic variants. Dedicated tools, such as SpliceAI [66] and Pangolin [67], have been developed to predict variant effects on RNA splicing. Newer models such as AlphaGenome [68], aim to predict the effects of sequence variation on a broader range of molecular properties, including gene expression and regulatory activity. However, these predictions provide indirect evidence of variant effects and should not be interpreted as definitive evidence of pathogenicity. Their reliability depends on the variant type and the underlying disease mechanism; predictions may be more informative for some loss-of-function mechanisms than for gain-of-function or dominant-negative effects, which are not necessarily captured by conventional deleteriousness scores. Computational predictions of variant effects should also be distinguished from assessments of pathogenicity, as predicting a molecular consequence does not, by itself, establish whether a variant causes disease.

Family studies can provide additional evidence. Segregation of a variant with disease across multiple affected and unaffected relatives can support pathogenicity, although this interpretation must account for reduced penetrance and variable expressivity [69]. If available, well-validated functional studies that take the variant out of the context of the individual’s genome and demonstrate its ability to reproduce expected molecular, cellular and/or phenotypic consequences are another useful form of evidence for pathogenicity, though come with their own interpretational caveats. The relevance of the assay to the disease mechanism, its validation, reproducibility, appropriate positive and negative controls, and its ability to distinguish established pathogenic and benign variants should all be considered [70]. Studies in patient cells may also be informative, though at present can formally only be considered one contribution to overall phenotypic scoring.

Thus, when a VUS is identified, it is important to consider the totality of available evidence—including the gene–disease relationship, phenotype, inheritance pattern, population frequency, computational predictions, and family data—while recognizing the limitations of each individual evidence.

## Management of Unresolved Cases

It is important to note that given the current limitations, a negative genetic result should not exclude a GID when clinical suspicion remains high. In such cases, complementary genomic, transcriptomic, and functional studies become critical to establishing a diagnosis [71].

Beyond DNA-based analysis, transcriptomic and functional approaches are increasingly important in unresolved cases. Although RNA sequencing is not yet routinely available in most clinical diagnostic laboratories, it can be used to identify aberrant splicing or other expression-level differences if performed in a relevant and accessible cell type. These approaches are particularly useful for clarifying VUSes and for detecting the functional impact of intronic or regulatory variants [72]. Similarly, patient-derived studies of protein expression, signaling pathways, cytokine responses, or cellular phenotypes can provide valuable supporting evidence to aid timely clinical decision-making.

In vitro experimental models, including overexpression systems or genome-edited cellular models, may then provide more direct functional evidence required to establish disease causality. When these complementary approaches identify a plausible candidate variant or molecular mechanism, reverse phenotyping—the systematic re-evaluation of the patient’s clinical, immune, and laboratory features in light of the genetic finding—may reveal previously unrecognized manifestations that strengthen the genotype–phenotype correlation and support variant interpretation.

Periodic reanalysis of sequencing data has emerged as a powerful strategy to increase diagnostic yield over time. As new disease genes and mechanisms are continuously identified, previously negative datasets may eventually reveal a molecular diagnosis upon reinterpretation [42]. Systematic reanalysis, supported by routinely updated annotation databases, is therefore an essential component of modern genomic diagnostics in GID.

Finally, data-sharing initiatives have become valuable tools for gene discovery and variant interpretation in rare disorders. Platforms such as Matchmaker Exchange [73] including GeneMatcher [74], PhenomeCentral [75] or RD-Connect [76], facilitate the identification of additional unrelated individuals with variants in the same gene or overlapping phenotypes, thereby strengthening gene–disease associations and enabling variant reclassification in previously unresolved cases. These collaborative frameworks may be particularly useful when assessing novel candidate genes or unresolved variants in rare and clinically challenging cases.

## Conclusion

The genetic evaluation of patients with suspected GIDs should be viewed as an iterative, multidisciplinary process integrating clinical assessment, immune phenotyping, genomic analysis, and functional investigation. Careful integration of clinical and immunologic features remains fundamental to identifying patients most likely to benefit from genetic testing and selecting the most appropriate diagnostic strategies. As genome technologies continue to evolve, their greatest clinical value will depend not only on improved technical capabilities but also on thoughtful interpretation within the patient’s biological and clinical context. Even when initial testing is inconclusive, complementary transcriptomic and functional approaches, periodic data reanalysis, reverse phenotyping, and collaborative data sharing continue to enhance diagnostic performance. Ultimately, combining mechanism-based clinical reasoning with an integrated genomic approach will support individualized clinical management for patients with immune dysregulation.

## Summary Sentence

Genetic testing for genetically driven immune diseases is most effective when guided by careful clinical and immunologic evaluation, appropriate test selection, and integrated interpretation of molecular findings.

## Key References

- Kindle G, Alligon M, Albert MH, Buckland M, Edgar JD, Gathmann B, et al. Inborn errors of immunity: manifestation, treatment, and outcome - an ESID registry 1994-2024 report on 30,628 patients. J Hum Immun. 2025 Jul 17;1(3):e20250007.
- Chan AY, Torgerson TR. Primary immune regulatory disorders: a growing universe of immune dysregulation. Curr Opin Allergy Clin Immunol. 2020 Dec;20(6):582–90.
- Gans MD, Eisenberg R. Immune Dysregulation Leading to Autoimmunity. Primary and Secondary Immunodeficiency. 2021;221–53.
- Poli MC, Aksentijevich I, Bousfiha AA, Cunningham-Rundles C, Hambleton S, Klein C, et al. Human inborn errors of immunity: 2024 update on the classification from the International Union of Immunological Societies Expert Committee. J Hum Immun. 2025 May 5;1(1):e20250003.
- Klangkalya N, Fleisher TA, Rosenzweig SD. Diagnostic tests for primary immunodeficiency disorders: Classic and genetic testing. Allergy Asthma Proc. 2024 Sep 1;45(5):355–63.
- Caballero-Oteyza A, Crisponi L, Peng XP, Yauy K, Volpi S, Giardino S, et al. GenIA, the Genetic Immunology Advisor database for inborn errors of immunity. J Allergy Clin Immunol. 2024 Mar;153(3):831–43.

## Data Availability

No datasets were generated or analysed during the current study.
